# Placenta specific gene targeting to study histone lysine demethylase and androgen signaling in ruminant placenta

**DOI:** 10.1590/1984-3143-AR2020-0069

**Published:** 2020-06-11

**Authors:** Taylor Kimberly Hord, Agata Maria Parsons Aubone, Asghar Ali, Hayley Nicole Templeton, River Evans, Jason Edward Bruemmer, Quinton Alexander Winger, Gerrit Jerry Bouma

**Affiliations:** 1 Animal Reproduction and Biotechnology Laboratory, Department of Biomedical Sciences, Colorado State University, Fort Collins, CO, USA; 2 Animal Reproduction and Biotechnology Laboratory, Department of Animal Sciences, Colorado State University, Fort Collins, CO, USA

**Keywords:** KDM1A, androgen receptor, trophoblast cells, placenta

## Abstract

Reproductive efficiency is critically dependent on embryo survival, establishment of a successful pregnancy and placental development. Recent advances in gene editing technology have enabled investigators to use gene knockdown and knockout approaches to better understand the role of hormone signaling in placental function and fetal growth and development. In this review, an overview of ruminant placentation will be provided, including recent data highlighting the role of histone lysine demethylase 1A and androgen signaling in ruminant placenta and pregnancy. Studies in ruminant placenta establish a role for histone lysine demethylase 1A in controlling genetic networks necessary for important cellular events such as cell proliferation and angiogenesis, as well as androgen receptor signaling during early placentation.

## Introduction

Profitability in livestock industry is directly related to the ability of the dam to produce healthy offspring in the shortest possible time. Establishment of pregnancy at a younger age, they produce milk sooner, have shorter calving intervals and generate more replacements. The main reason cows are removed from the production herd is the inability to conceive or maintain pregnancy. Pregnancy loss has been reported to vary from 3% to 42% in beef and dairy cattle (Gatea et al., 2018). Reproductive efficiency in livestock is critically dependent on fertility and embryo survival. In ruminants, fertilization itself is generally high (~90%), however embryonic losses can be quite significant and as high as 40% for heifers or 60% for high-producing dairy cows (Diskin and Morris, 2008). Establishment of pregnancy is dependent on early signals from the embryo to the mother and formation of a placenta. In fact, miscommunication between the growing embryo and the endometrium is thought to be mainly responsible for many early embryo and fetal loses, and a failure to maintain pregnancy (Spencer, 2013).

In cows and sheep, the embryo elongates dramatically prior to attachment to the uterine wall and secretes interferon tau (IFNT), and placental development soon commences (reviewed in Spencer et al., 2004; Spencer, 2013). IFNT, the signal for maternal recognition of pregnancy, is critical to prevent prostaglandin F2 alpha (PGF2α) secretion from the endometrium and thus luteolysis, thereby maintaining the corpus luteum and continued progesterone and estradiol secretion. Steroid hormones play an essential role in the maintenance and progression of pregnancy, fetal development and growth, and parturition. Although the placenta itself is recognized as an endocrine organ, most notably by its secretion of progesterone, androgens and estrogens, it is also a target of these steroid hormones which play an important role in placental development and function. Therefore, regulation of sex steroid signaling is critical for placental cell development and differentiation. Recently, histone lysine demethylase 1A (KDM1A) has emerged as a critical regulator of gene expression and function in trophoblast cells by interacting with and controlling steroid hormone signaling. In this review, an overview is provided on the role of KDM1A and androgen receptor (AR) in placental development by controlling trophoblast cell differentiation, with a focus on ruminant placentation.

## Early placental development in ruminants (ewe and cow)

Placentation in mammals can be classified by morphology (i.e., diffuse, zonary, discoid, cotyledonary), or physiologically more relevant by histological structure and organization of the layers separating maternal and fetal circulation (i.e., epitheliochorial, syndesmochorial, endotheliochorial, hemochorial) (reviewed in Chavatte-Palmer and Tarrade, 2016). Ruminant placenta is classified by morphology as cotyledonary, and histologically as epitheliochorial, syndesmochorial.

In the ewe, the developing embryo enters the uterus at day 4 as a morula, developing into a blastocyst by day 6 (Spencer et al., 2004). By day 8, the blastocyst hatches from the zona pellucida and is located in the ipsilateral uterine horn (Spencer et al., 2004; Rowson and Moor, 1966). It undergoes a period of rapid cell proliferation and elongation, growing into the contralateral horn by day 13 in a singleton pregnancy (Rowson and Moor, 1966). During this period of elongation the extra-embryonic membranes (chorion and yolk sac) form prior to implantation (Spencer et al., 2004; Renfree, 1982; Guillomot et al., 1993; Carson et al., 2000). Rapid elongation of the ovine conceptus continues until day 16, when it adheres to the uterine epithelium (Spencer et al., 2004; Rowson and Moor, 1966; Grazul-Bilska et al., 2011).

**Figure 1:**
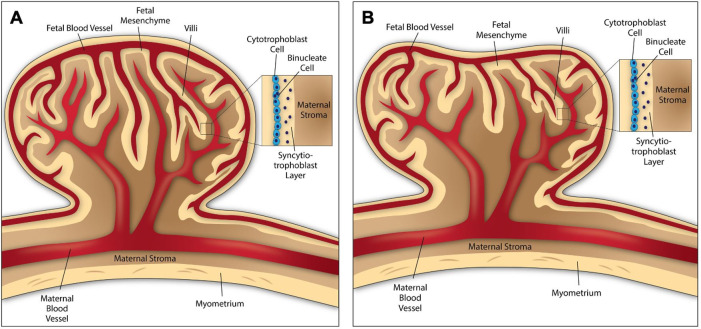
Schematic illustrating cow (A) and sheep (B) placentomes.

Early placentation in ruminants starts when elongated embryos attach at discrete sites to the uterine wall called caruncles, which are aglandular sites along the uterine epithelium. Some comparative characteristics between sheep and cow embryos and placentation are highlighted in Table 1. Between days 14 and 16 in sheep, when the conceptus is rapidly elongating and beginning to adhere to the endometrial luminal epithelium, binucleate trophoblast (BNC) cells begin to differentiate from the mononuclear trophoblast cells through consecutive nuclear divisions without cytokinesis (Spencer et al., 2004). At around gestational day 16, the sheep embryo has developed into a ~25cm long structure due to extensive proliferation of the trophectoderm. In ruminants, the placentome consists of a fetal cotyledon containing the chorion (trophoblast cells) and a maternal caruncle originating from the endometrium. In the cow, placentomes form a convex structure, while in the sheep they appear concave (Figure 1). BNC fusion with uterine luminal epithelial cells leads to formation of a multinucleated syncytiotrophoblast layer. More recently in sheep it has been proposed that mononuclear trophoblast cells can also form multinucleated trophoblast giant cells (TGC) (Seo et al., 2019), which are believed to remove uterine luminal epithelial cells, and fuse to form the syncytial trophoblast layer. Syncytial plaques develop specifically within the placentome, covering the surface of caruncles for nutrient and gas exchange (Spencer et al., 2004).

**Table 1:** Comparison between cow and sheep placentation.

	**Bovine**	**Ovine**
**Gestation Length**	279-287 Days	144-152 Days
**Placentome Shape**	Cotelydonary, convex	Cotelydonary, concave
**Average Placentome Amount**	75-125	75-125
**Blastocyst Hatching**	Days 7-10	Days 7-8
**Conceptus Growth**	Day 15: 1-2 mm Days 18-19: 10-20 cm	Day 12: 10-22 mm Day 14: 10 cm Day 17: 25 cm
**Implantation Attachment Time**	Beginning Day: 22 Completion Day: 40	Beginning Day: 15-16 Completion Day: 22-28
**Total Placentome Weight**	• Rate of increase decreases with time, with a significant increase between 60-190 days after conception • Up to 5000 g	Increases throughout pregnancy up to 579.4 g
**Average Placentome Weight (g)**	50 g	7.91
**Total Caruncular Weight (g)**		101.6
**Total Cotyledonary Weight (g)**		291.5
**Luteal Progesterone**	Required throughout gestation	Placenta takes over production around day 50-60
**Binucleate Trophoblast Cells**	• Formed from mitotic divisions of mononucleate cells that do not undergo cytokinesis • Produce bovine placental lactogen	• Formed from mitotic divisions of mononucleate cells that do not undergo cytokinesis between days 14-16 • Produce ovine placental lactogen • Express placental lactogens and pregnancy-associated glycoproteins
**Trophoblast Giant Cells/Multinucleated Trophoblast Cells**	• Involved in syncytialization • Formed through mononuclear trophoblast cell fusion • Migrate and fuse with uterine epithelium forming syncytium • Function in gas and nutrient exchange and hormone production	• Involved in syncytialization • Formed through mononuclear trophoblast cell fusion • Insert between the uterine epithelial cells that are simultaneously undergoing apoptosis • Fuse with each other to form trophoblast syncytial layer • Function in gas and nutrient exchange and hormone production

These early events are critical for the development of a proper functioning placenta and establishment of pregnancy. The development of novel approaches to study gene function specifically in the placenta has allowed for new insight into the molecular events underlying placental development.

## Studying gene function in the ruminant placenta

**Figure 2:**
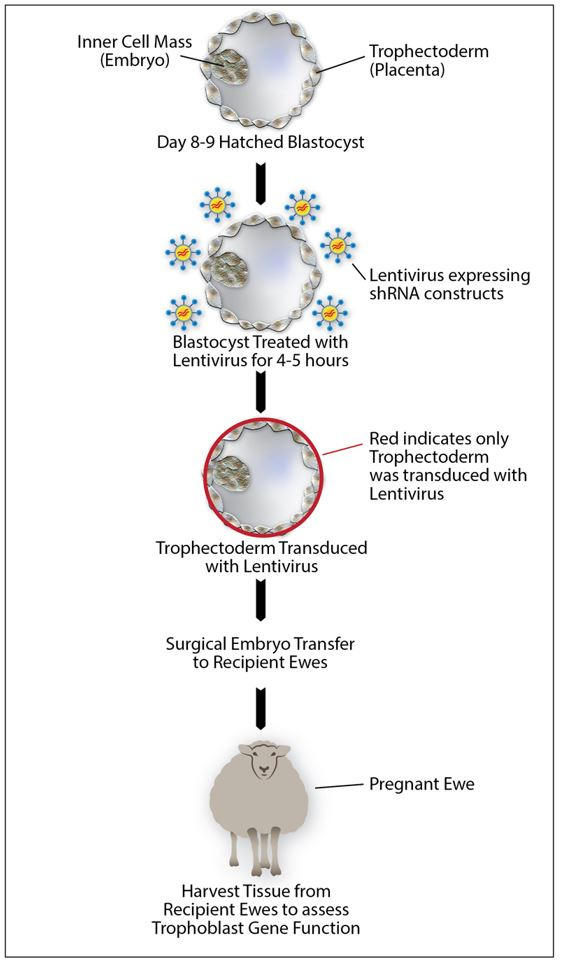
Illustration of a trophectoderm specific gene editing approach used to study gene function in sheep placenta.

The ability to specifically target gene expression in the trophoblast layer (trophectoderm) was first described in mice (Georgiades et al., 2007). In this study, Georgiades and colleagues demonstrated that short-term transduction of mouse blastocysts with a green fluorescent protein (GFP) transgene led to expression of GFP only in the trophectoderm, and subsequently in the whole placenta, but not in the embryo or fetus. This technology has now successfully been used to study several genes in sheep placenta, including PRR15 (Proline-Rich 15) (Purcell et al., 2009), CSH (Chorionic somatomammotropin hormone) (Baker et al., 2016), and LIN28 (Ali et al., 2020). The general approach to edit gene function specifically in the trophectoderm (Figure 2) involves collecting day 8-9 hatched blastocysts. At this time, the trophoblast layer is directly exposed to the virus, and before loss of the Rauber’s layer which covers and shields the epiblast (Van Leeuwen et al., 2020). The blastocysts are transduced with lentivirus expressing shRNA target constructs for 4-5 hours. Single transduced embryos are then transferred to recipient ewes, and day 16 blastocysts can be collected before attaching to the endometrium to assess trophoblast gene function to study embryo elongation. Near term, fetuses and placenta can be collected to elucidate effects on placental development and function and fetal growth and development. Combined with the continued advances in gene editing tools such as CRISPR-cas9, unique opportunities now exist to study gene function (knock-out, knock-down, knock-in, overexpression, etc.) and hormone signaling specifically in ruminant placentas. Not only could this benefit the livestock industry by providing much needed insight into the factors necessary for early pregnancy establishment and maintenance, but also be of great value to human biomedicine with the development of large animal models to study human pregnancy. In fact, sheep have been used extensively as animal models to study human pregnancy complications (Barry and Anthony, 2008).

## Androgens during pregnancy in livestock

**Figure 3:**
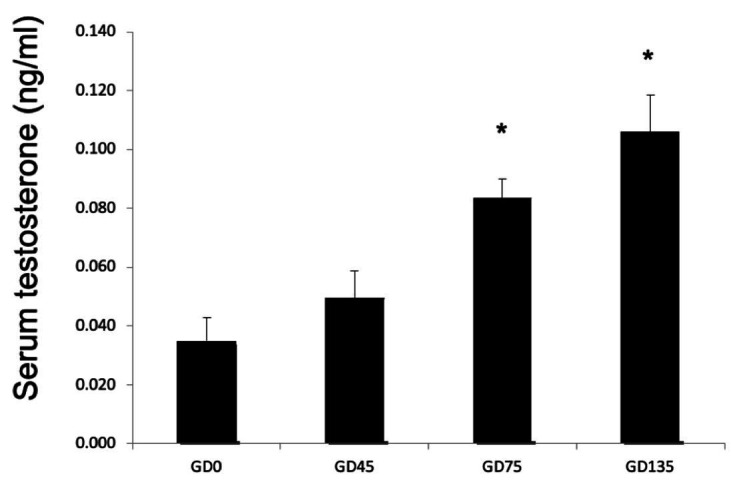
Serum testosterone levels in maternal sheep blood during pregnancy. GD: gestational day. *P < 0.05.

Although the placenta is a well-recognized endocrine organ, the role of steroid hormones, particularly androgens, in placental development or function are less clear. This is surprising considering maternal serum level of testosterone increases during normal pregnancy (Figure 3). Transcripts of *HSD3b, CYP11A1,* and *CYP17* are all present in ruminant placentomes (Vanselow et al., 2004) and synthesis of androgens are localized to cotyledonary trophoblast cells (Nguyen et al., 2012). In cow and sheep placentomes, AR is present in trophoblast cells, cotyledonary stromal cells, and caruncular epithelial and stromal cells (Khatri et al., 2013; Cleys et al., 2015). In cows, prior to gestational day 272 AR appeared to only localize to invasive TGCs and fetal-maternal hybrid cells, formed by fusion of invasive TGCs with caruncular epithelial cells (Khatri et al., 2013).

Very little is known about the role of androgens in placental abnormalities and pregnancy disorders in ruminants. In humans, abnormal maternal serum testosterone concentration is associated with metabolic and placental disorders, such as IUGR, which negatively impacts fetal development and leads to embryo losses. Sheep have been used extensively as a model to study polycystic ovarian syndrome (PCOS). Female sheep fetuses exposed to testosterone during early to mid-gestation exhibited ovarian dysfunction and reproductive changes in the hypothalamus-hypophysis-gonadal axis after puberty (Forsdike et al., 2007). These ewes experienced metabolic characteristics similar to PCOS in women. Furthermore, gestational testosterone treatment in sheep induces distinct changes in the placental milieu. Pregnant ewes treated with testosterone starting at gestational day 30 developed placental lipotoxicity and collagen deposition. This suggests that androgen-induced lipid accumulation can lead to tissue damage and fibrosis as well as impaired placental function. This study also demonstrated that an increase in collagen deposition due to excess androgen levels offers a potential mechanism for development of fibrotic lesions in conditions of placental deficiency (Kelley et al., 2019).

Prenatal testosterone treatment increases placental expression of vascular endothelial growth factor A (VEGFA), which is secreted in response to tissue hypoxia and endothelial damage. This coincides with several studies demonstrate that an increase in plasma testosterone is implicated in placental vasculopathies, possibly due to the increased vascular tone of spiral arteries (Maliqueo et al., 2016; Kumar et al., 2018). Moreover, prenatal androgenization leads to altered placentome morphology with increased numbers of type D (more interdigitated fetal villi, heightened cotyledon proliferation and increased vascular density) compared to type A placentomes. Prenatal androgenization induces reduced fetal weight, suggesting that increased androgen signaling dysregulates fetal development possibly through impaired nutrient transport (Cleys et al., 2015). Gestational testosterone and dihydrotestosterone exposure produced low-birth-weight female offspring and these actions appear to be through impaired AR signaling (Beckett et al., 2014).

Detrimental effects of prenatal androgenization and induced altered uterine environment on fetal developmental programming of reproductive tissues have been well-documented. Some of these long-term changes in female offspring originate during fetal life and involve an increase in AR in granulosa, theca and stromal compartments of fetal ovaries. In addition, post-pubertal ovaries exhibited increased AR expression in large preantral and antral follicles, while reduced expression of AR was evident in stromal cells (Padmanabhan et al., 2010). In male offspring, testosterone excess during fetal development also impacts testicular development, mainly characterized by an increase in Sertoli cell number (Rojas-García et al., 2013). Ultimately both female and male offspring demonstrated reduced fertility in adulthood. Finally, prenatal androgenization leads to decreased global DNA methylation in placentomes, and increased placental expression of AR (Cleys et al., 2015), and gene expression analysis has demonstrated mis-expression on various genes involved with epigenetic regulation of gene function, including DNA methyltransferases and histone lysine demethylases, including KDM1A.

## KDM1A and gene regulation in trophoblast cells placental development and function

A nucleosome contains DNA wrapped around a histone octamer. Histone octamers have four types of histones; H2A, H2B, H3, and H4. Histone lysine methyltransferases are a group of enzymes that methylate lysines in histone tails. Histone lysine methylation leads to a variety of biological processes like transcriptional activation and repression. Histone lysine methyltransferases can methylate up to three methyl groups on lysine histone tails (Nottke et al., 2009). Conversely, there are several different histone lysine demethylases that remove methyl groups and each serve a specific biological role in regulating chromatin and epigenetic factors. KDM1A is a histone lysine demethylase that demethylates mono- and di-methylated lysine 4 and 9 on H3 (H3K4me1/2 and H3K9me1/2). Demethylation of H3K4me1/2 causes transcriptional repression, whereas demethylation of H3K9me1/2 generally causes transcriptional activation. KDM1A contains a flavin adenine dinucleotide (FAD) dependent amine oxidase domain that is responsible for histone demethylation. This oxidase enzyme uses FAD to help catalyze amine oxidation to create iminium ions. Water then joins with the iminium ion to release formaldehyde resulting in a mono-methylated lysine. The mono-methylated lysine undergoes the same reaction to become completely demethylated (Husmann and Gozani, 2019).

**Figure 4:**
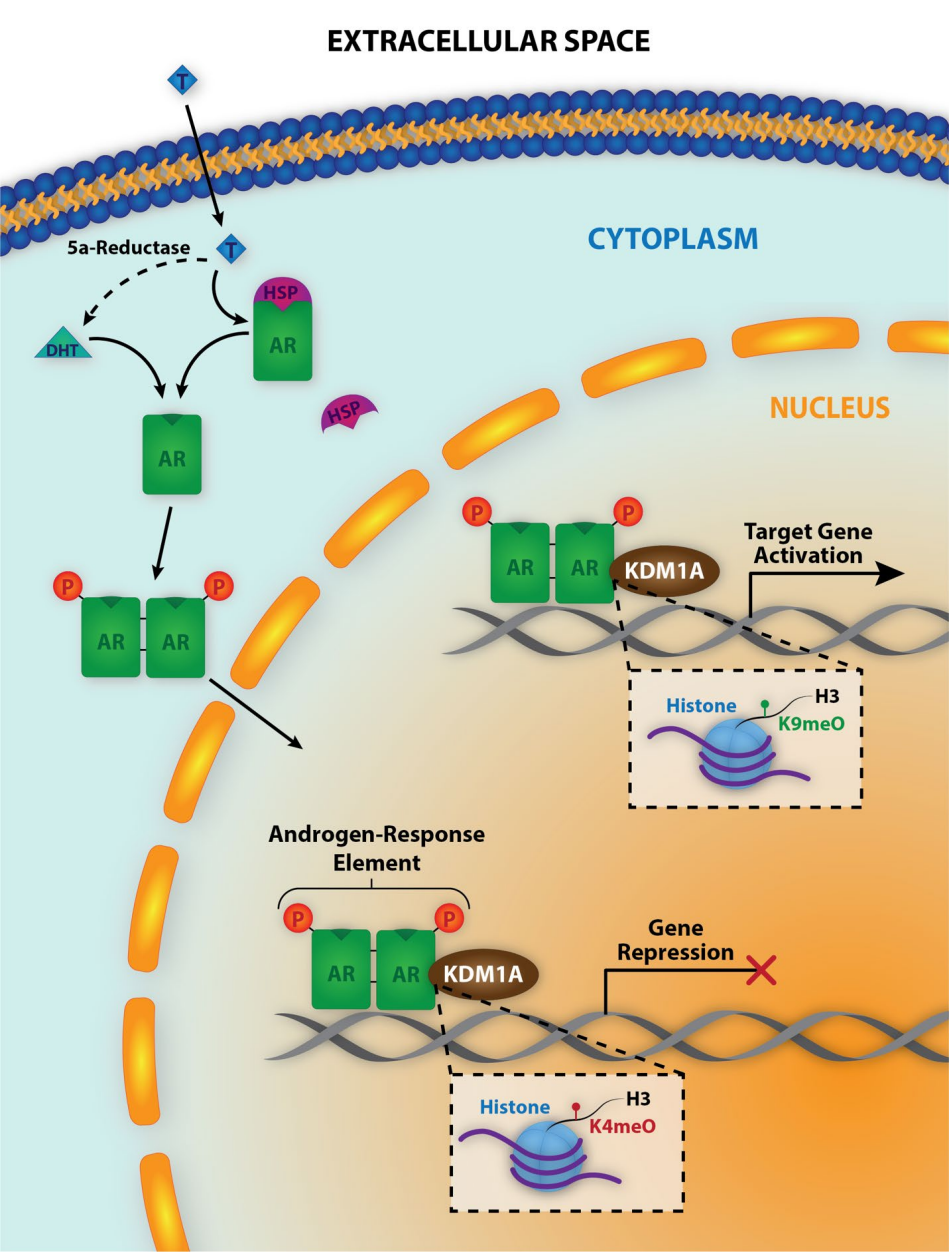
KDM1A and AR signaling in trophoblast cells. T: testosterone, DHT: dihydrotestosterone, HSP: heat shock protein, P: phosphorylated, H3: histone 3

KDM1A is a subfamily of amine oxidases which play a vital role in regulating transcription of both normal and disease signaling pathways (Burg et al., 2015). Evidence on the existence of KDM1A first came in 2004 (Shi et al., 2004), and subsequent studies have demonstrated its involvement with cancer and tumorigenesis. Overexpression of KDM1A in neuroblastoma is possibly linked to impaired KDM1A-silencing of microRNAs (Althoff et al., 2013). Inhibiting KDM1A in neuroblastoma cells increased H3K4 methylation, decreased cell proliferation, and reduced tumor growth [9]. Histone lysine demethylases also play a role in regulating nuclear hormone signaling by binding to and interacting with androgen receptor (AR) and estrogen receptors (ESR), and activating or inhibiting AR and ER responsive genes (Wissmann et al. 2007; Garcia-Bassets et al., 2007) (Figure 4). KDM1A has also been correlated to regulating breast cancer, and studies revealed that inhibiting KDM1A decreases proliferation of breast cancer cells (Lim et al., 2010). Similarly, prostate cancer has also been thought to be linked to KDM1A due to KDM1A-mediated transcriptional regulation of AR, and KDM1A inhibition decreases prostate cancer proliferation (Zhang et al., 2009; Willmann et al., 2012).

Considering the many similarities in cellular processes such as proliferation, migration and invasion between tumor progression and placental development, it is not surprising KDM1A also is present in placenta (Cleys et al., 2015). Immunostaining revealed KDM1A in human first trimester placenta localized to the syncytiotrophoblast and villi stromal cells. In sheep placentomes, immunofluorescence revealed KDM1A localizes to the nuclei of trophoblast cells. As in cancer cells, KDM1A binds to AR and ESR1 in trophoblast cells (Cleys et al., 2015), and therefore is thought to play a role in regulating AR and ESR1 signaling in the placenta. Considering the fact that AR and ESR1 play a role in cell proliferation and angiogenesis in cancer (Chang et al., 2014; Pequeux et al., 2012), it is likely they exert similar functions in placentas.

Placental development relies on angiogenic factors to allow vascular remodeling in maternal spiral arteries to increase blood flow to the placenta. In ruminants, vascular remodeling occurs in maternal tissue (caruncles) and capillary branching increases in fetal tissue (cotyledon) (Reynolds et al., 2005). In intra-uterine growth restriction (IUGR) pregnancies placental abnormalities occur and abnormal blood flow to the fetus in these pregnancies causes impaired fetal growth (Regnault et al., 2002; Reynolds et al., 2005). IUGR pregnancies were shown to have decreased VEGFA along with its receptors (VEGFR1 and VEGFR2) (Regnault et al., 2003). The ovine VEGFA promoter contains both androgen and estrogen response elements (ARE and ERE, respectively). Of interest here is the observation that AR and KDM1A interact with each other and bind the same ARE in the VEGFA promoter region in sheep placentomes (Cleys et al., 2015), suggesting a role for KDM1A in regulating AR-mediated VEGFA expression.

Previous work in mice demonstrated KDM1A is necessary for placental development. KDM1A knockout mice, with a specific deletion of KDM1A in trophoblast stem cells, demonstrated significantly reduced trophoblast development compared to wildtype mice (Zhu et al., 2014). Absence of KDM1A resulted in early embryonic lethality by gestational day 8.5, demonstrating KDM1A is necessary for placental development (Zhu et al., 2014). The knockout embryos also displayed morphological abnormalities such as smaller sizes and reduced trophoblast development compared to wildtype mice. In addition, a recent study using human choriocarcinoma (BeWo) cells revealed a role for KDM1A in cytotrophoblast differentiation into syncytiotrophoblasts by recruiting transcription factor GATA2 and stimulation of hCG production (Milano-Foster et al., 2019).

**Figure 5:**
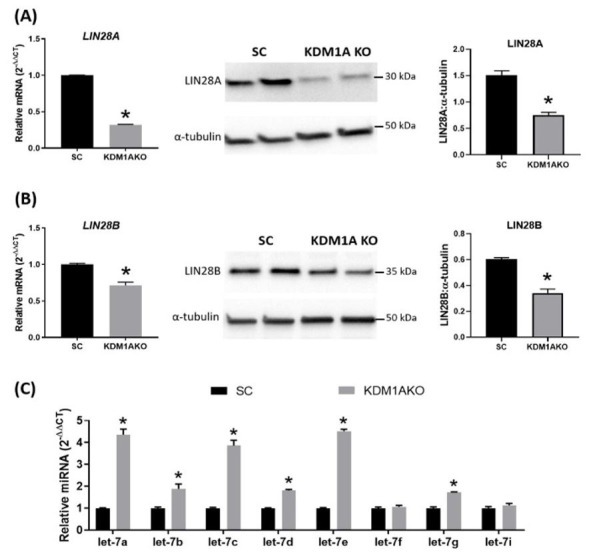
LIN28 and Let7 microRNAs in KDM1A knockout ACH-3P trophoblast cells. (A) LIN28A mRNA and protein amounts in KDM1A knockout ACH-3P trophoblast cells; (B) LIN28B mRNA and protein amounts in KDM1A knockout ACH-3P trophoblast cells; (C) Relative amounts of Let7 microRNA family members in KDM1A knockout ACH-3P trophoblast cells. SC: scramble control. Asterisk indicates P < 0.05 (Student’s t-test).

**Figure 6:**
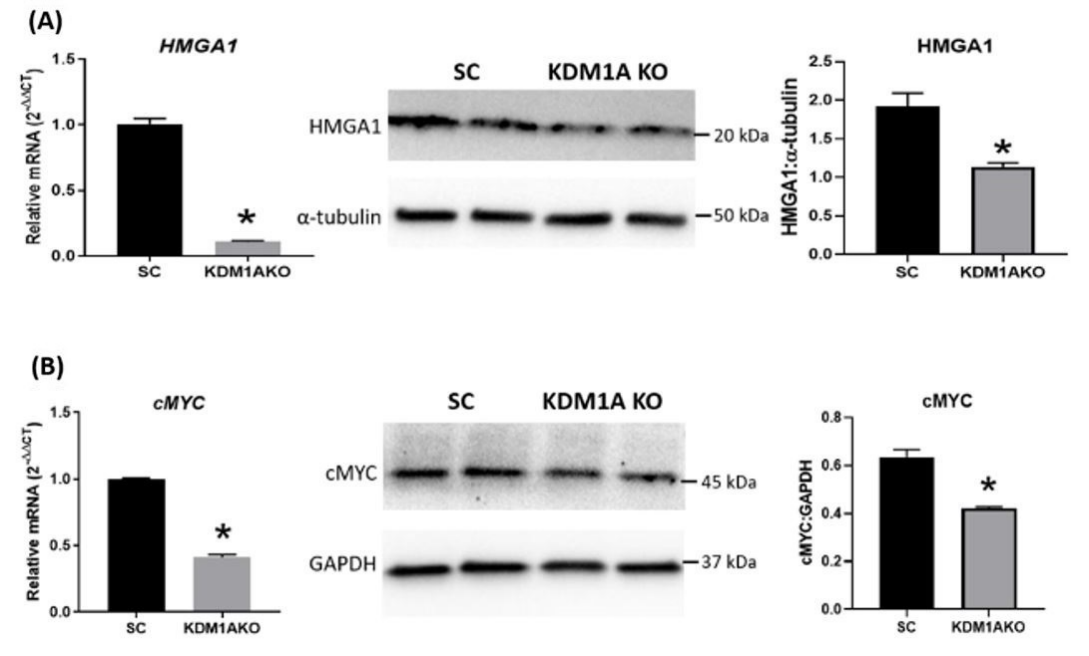
Relative amount of HMGA1 mRNA and protein (A), and cMYC mRNA and protein (B) in KDM1A knockout ACH-3P trophoblast cells. SC: scramble control. Asterisk indicates P < 0.05 (Student’s t-test).

To further study the role of KDM1A in regulating trophoblast gene expression and function, first trimester trophoblast cells were transduced with a KDM1A CRISPR-cas9 lentiviral gene targeting construct to knockout KDM1A. Subsequent in vitro gene expression analysis revealed that KDM1A knockout trophoblast cells had significantly reduced levels of the RNA binding protein LIN28 (Figure 5). Recent work revealed that LIN28 (A and B) regulates several important cell proliferation associated genes in sheep placenta, including IGF2BP1, IGF2BP2, IGFBP3, HMGA1, ARID3B and c-MYC (Ali et al., 2020), and resulted in significantly impaired conceptus elongation (Ali et al., 2020). These changes in gene expression and conceptus elongation possibly involves the LIN28-Let-7 axis, in which decreased LIN28 leads to a concomitant increase in let-7 microRNAs, which in turn regulate many of these cell proliferation associated genes. KDM1A knockout in trophoblast cells *in vitro* resulted in significantly lower levels of LIN28, and a subsequent increase in let-7 microRNAs (Figure 5). Furthermore, similar to LIN28 knockdown, a significant decrease in HMGA1 and c-MYC was observed (Figure 6). Moreover, our previous work demonstrated LIN28 also regulates AR expression, possibly through let-7c in trophoblast cells, and inhibiting LIN28 resulted in reduced AR expression and increased trophoblast cell differentiation (McWhorter et al., 2019). Therefore in trophoblast cells, KDM1A possibly plays an important role in regulating AR and other target genes directly or through its interaction with the LIN28-Let-7 axis.

Overall these data indicate a very complex genetic network regulating gene function in trophoblast cells that is necessary to maintain a balance between trophoblast cell proliferation and differentiation. Recognizing the ability of KDM1A to interact with and modulate methyl groups at different histone lysine tails highlights its potential role as one of the master regulators in gene regulation and functioning of trophoblast cells.

## Future perspectives

Currently several experiments are ongoing to study KDM1A and AR specifically in the ruminant placenta. The continued development of novel gene targeting techniques have now enabled unique opportunities to modulate gene expression and function specifically in the ruminant placenta, opening new avenues to uncover the complex genetic signaling pathways required for placental development and function, and establishment of a successful pregnancy. Thus far, a variety of studies have demonstrated the critical role for AR signaling in placentation and pregnancy, and KDM1A appears to be an important modulator of AR function. Future experiments will further highlight its potential role in placental angiogenesis and pregnancy maintenance. These data are necessary to obtain better insight into possible mechanisms of early embryo losses in ruminants, and could provide for novel avenues to prevent, alleviate and/or treat pregnancy complications.
